# Evaluating the Impact of Reminiscence Therapy on Cognitive and Emotional Outcomes in Dementia Patients

**DOI:** 10.3390/jpm14060629

**Published:** 2024-06-13

**Authors:** Nobuhiko Yanagida, Takumi Yamaguchi, Yuko Matsunari

**Affiliations:** 1 School of Health Sciences, Kagoshima University, Kagoshima 890-8544, Japan; yanagida@health.nop.kagoshima-u.ac.jp (N.Y.); matsuy@health.nop.kagoshima-u.ac.jp (Y.M.); 2 School of Nursing, Tokyo Medical University, Tokyo 160-8402, Japan

**Keywords:** reminiscence therapy, dementia, elderly care, cognitive function, EEG measurements

## Abstract

This study examines the impact of reminiscence therapy on cognitive and emotional well-being in institutionalized older patients with dementia. Conducted at the Long-Term Care Health Facility for the Elderly, the research involved 34 participants who underwent therapy sessions that included personalized discussions of past experiences. Using physiological markers such as electroencephalography alpha and beta waves, along with psychological measures such as the Hasegawa Dementia Scale—Revised, the study aimed to quantify the effects of the therapy. Although the results indicated positive correlations between alpha and beta waves, suggesting enhanced relaxation and cognitive engagement, improvements in Hasegawa Dementia Scale—Revised scores were not statistically significant, pointing to variability in therapeutic effectiveness among patients. Despite these mixed outcomes, the findings support the potential of reminiscence therapy as a non-pharmacological intervention to improve the quality of life of dementia patients, though they also underscore the necessity for further research to refine therapy protocols and enhance applicability.

## 1. Introduction

Dementia is a significant global health concern. It is of particular concern in Japan, where the population is aging rapidly. According to recent estimates, the number of individuals diagnosed with dementia has risen steadily in Japan, emphasizing the growing need for effective care and treatment modalities for this population [1,2]. Dementia manifests as a spectrum of cognitive and behavioral symptoms, which can be classified into core symptoms such as memory loss, language impairment, and a decrease in cognitive function, as well as peripheral symptoms including psychological and psychiatric changes [3,4]. Given the progressive nature of these symptoms, many individuals eventually require institutional care. The transition to such care not only affects the quality of life of the patients but also poses various challenges for healthcare providers in managing the symptoms effectively [5].

One therapeutic approach that has been revisited over the years is reminiscence therapy, first proposed by Robert Butler in the 1960s [6,7]. Reminiscence therapy involves discussion of past experiences, individually or in a group setting, facilitated by trained staff using tangible prompts to evoke memories [8]. Research suggests that reminiscence therapy can have beneficial effects on the psychological well-being of dementia patients, potentially reducing symptoms such as depression and anxiety, as well as improving cognitive function [9].

Furthermore, physiological assessments conducted during reminiscence therapy sessions indicated significant changes in biological markers, including alterations in brain wave activities (specifically, decreases in both alpha [α] and beta [β] waves) [10]. Salivary levels of amylase, a stress marker, have also been reported to decrease during these sessions, providing biochemical evidence of its stress-reducing effects [11]. Additionally, cognitive functions, as measured by tools such as the Hasegawa Dementia Scale, have shown improvements post-therapy [12]. The importance of investigating the effects of reminiscence therapy is underscored by its potential to improve the daily lives of dementia patients, many of whom spend substantial portions of their day in states of agitation or unrest. Integrating reminiscence therapy has the potential not only to improve physiological and psychological health but also to enhance overall care quality [13]. Reminiscence therapy, therefore, has possibility to offer a non-pharmacological intervention that might reduce the reliance on medications, which often come with significant side effects, especially in older adults. Previous studies have demonstrated the positive impact of reminiscence therapy on both psychological and physiological parameters. For example, Saletu et al. (1991) and Tanaka et al. (2007) highlighted improvements in EEG patterns and cognitive functions following reminiscence therapy [10,12]. However, these studies primarily focused on observational data and lacked rigorous experimental designs. This necessitates further research to confirm these findings through more structured methodologies and to explore the physiological underpinnings of reminiscence therapy’s effects more thoroughly.

The aim of this study is to evaluate the ability of group reminiscence therapy to improve severe dementia symptoms among older institutionalized patients, using physiological markers to quantify its impact. By analyzing EEG data, we intend to understand better how reminiscence therapy influences brain activity and its potential therapeutic benefits for dementia patients. This research contributes to our understanding of non-pharmacological interventions, which can significantly enhance the quality of life for those suffering from dementia.

## 2. Materials and Methods

**Figure 1:**
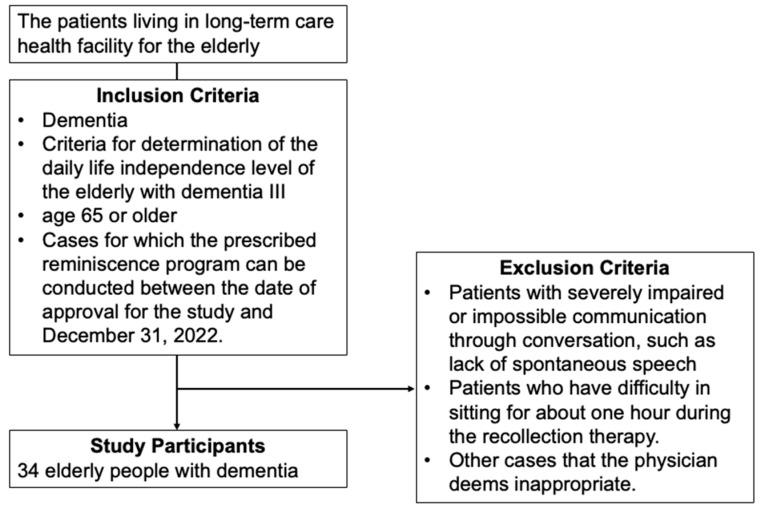
Enrollment of study participants.

This study enrolled 34 older adults with dementia who were residing at the Long-Term Care Health Facility for the Elderly. The participants included 15 males and 19 females, with a mean age of 87.88 years (SD 7.66). Eligibility criteria for the study included a diagnosis of dementia, specifically stage III dementia as classified by the Ministry of Health, Labour and Welfare guidelines, which describe symptoms and behaviors that interfere with daily life and occasional difficulty in communication, requiring nursing care [14]. All participants were aged 65 years or older and were admitted to the dementia ward because they could not be cared for at home. Exclusion criteria included patients with severely impaired communication abilities, those unable to sit for approximately one hour, and other cases deemed inappropriate by the attending physician (Figure 1).

The reminiscence therapy intervention consisted of eight components, including discussions about the place of birth, traditional events such as New Year’s Day and other festivals, school experiences, seasonal changes, materials associated with childhood and folk tools, life as a youth, and music from old Ministry of Education songs and nursery rhymes. These sessions were personalized to match the individual histories and preferences of each participant, ensuring high levels of engagement. An occupational therapist specializing in psychiatric rehabilitation conducted the sessions at the dementia ward from October 2022 to February 2023. Each group consisted of 7 to 10 participants, and sessions were held twice a week, totaling eight sessions over four weeks. Participants who were unwell or unwilling to attend a session were not compelled to participate.

Primary variables measured included simple electroencephalography (EEG) data, specifically β (13.5–30 Hz) and α (8–13 Hz) waves, obtained using the Brain Pro FM-939 electroencephalograph (FUTEC Electronics Co., Ltd.; Yokohama, Japan). Using a simple EEG, two FUTEK Sensor Pro SEN-PRO sensors are attached symmetrically to the left and right sides of the parietal region’s midline with conductive paste and measured for three minutes [15]. Brain waves are recorded to assess the state of relaxation. Measurements were taken before and after each session, as well as at baseline, pre-intervention assessment, and post-intervention assessment.

Cognitive function was assessed using the Hasegawa Dementia Scale—Revised (HDS-R), which evaluates memory, attention, calculation, and language abilities. The HDS-R consists of 11 items, scored between 0 and 1 or 0 and 2, with a total possible score ranging from 0 to 30. Scores below 19 indicate dementia, scores between 20 and 26 indicate mild cognitive impairment, and scores between 27 and 30 are considered normal. The HDS-R has high internal consistency and test-retest reliability [16,17]. Moreover, The HDS-R score of 20 points was selected as the cutoff for dividing patients into two groups based on its widespread use in clinical practice. This cutoff point is commonly employed to distinguish between mild cognitive impairment and more severe dementia. Utilizing this well-established threshold ensures consistency with previous research [16]. The Neuropsychiatric Inventory—Nursing Home Version (NPI-NH) was used to assess behavioral and psychological symptoms, consisting of 12 subdomains, such as delusions, hallucinations, agitation, depression, anxiety, euphoria, apathy, disinhibition, irritability, aberrant motor behavior, nighttime disturbances, and appetite changes. Each subdomain includes 3 to 5 questions, with total scores ranging from 0 to 144, based on the frequency and severity of symptoms [18,19]. HDS-R and NPI-NH were performed at baseline, pre-intervention assessment, and post-intervention assessment time points.

To analyze the data, the normality of continuous variables was assessed using the Shapiro–Wilk test and Q–Q plots [20]. None of the continuous variables followed a normal distribution, confirmed through both statistical tests and visual inspections. Consequently, a generalized linear mixed-effects model (GLMM) was used [21]. The GLMM included EEG β waves as the objective variable, with EEG α waves, age, NPI-NH score, HDS-R score, and the number of therapy sessions as fixed effects, and participant ID as a random effect. For analyzing pre-post changes in EEG, similar models were constructed with EEG α and β waves as the objective variables, pre-post EEG frequency and age as fixed effects, and participant ID as a random effect. Statistical analyses were conducted using R software (version 4.3.1), with a significance level set at *p* < 0.05.

Despite the small sample size, the use of GLMM was validated through references to Bolker et al. (2009) and Harrison et al. (2018) [22,23], which demonstrate the appropriateness and robustness of GLMM even in studies with limited sample sizes.

## 3. Results

### 3.1. Study Participants

We enrolled 34 patients (15 males and 19 females) in this study. The mean (standard deviation) age of the patients was 87.88 (7.66) years.

We observed that the mean EEG α wave value was 5.20 (1.35) before the intervention and 5.30 (1.48) after the intervention. Similarly, the mean EEG β wave value was 2.61 (0.98) before the intervention and 2.66 (0.99) after the intervention. The mean age of participants was 87.88 (7.73) years, and the mean NPI-NH and HDS-R scores were 15.65 (15.19) and 12.56 (5.78), respectively (Table 1).

### 3.2. Factors Related to EEG β Waves

The analysis revealed that the increase in EEG α waves significantly predicted the increase in EEG β waves, with an estimate of 0.61 (t = 10.64, *p* < 0.001). Other factors such as age (B = 0.02, t = 0.95, *p* = 0.349), NPI-NH score (B < 0.001, t = 0.06, *p* = 0.951), HDS-R score (B = −0.31, t = −0.96, *p* = 0.336), and the number of therapy sessions (B = −0.07, t = −1.04, *p* = 0.303) did not show a significant association with EEG β waves (Table 2).

### 3.3. Pre–Post EEG Changes

Table 3 and Table 4 show the data for pre–post EEG α and β wave changes. No significant changes were observed in the EEG wave values before and after the intervention for α waves (B = 0.12, t = 1.32, *p* = 0.188) or for β waves (B = 0.08, t = 1.00, *p* = 0.320).

The mean pre–post change in EEG α waves was estimated to be 0.12 (*p* = 0.188), indicating no significant change. Similarly, the number of sessions (B = −0.01, t = −0.30, *p* = 0.766) and age (B = 0.02, t = 0.96, *p* = 0.346) did not significantly predict changes in EEG α waves.

For EEG β waves, the mean pre–post change was estimated to be 0.08 (t = 1.00, *p* = 0.320), showing no significant change. The number of sessions also showed no significant change (B < 0.01, t = −0.07, *p* = 0.945). However, age significantly predicted changes in EEG β waves (B = 0.04, t = 2.92, *p* = 0.006).

The “Pre–post EEG changes” row specifically represents the average change in EEG waveforms from before to after the intervention. For both α and β waves, the estimates suggest slight increases, but these changes are not statistically significant.

The intercept values in both tables represent the baseline EEG wave values when other factors (like age and number of sessions) are held constant. The estimates for number of sessions and age indicate how these variables potentially influence EEG wave changes, with age showing a significant effect on β waves.

## 4. Discussion

This study aimed to evaluate the impact of reminiscence therapy on the physiological and psychological health of patients with dementia. Reminiscence therapy is a treatment that is expected to alleviate symptoms of dementia by encouraging patients to reflect on past experiences.

### 4.1. Detailed Analysis of the Correlation between EEG α and β Waves

The positive correlation observed between EEG α and β waves in this study is a significant finding for understanding the effects of reminiscence therapy in the treatment of dementia [24]. Generally, α waves are associated with relaxation, whereas β waves are linked to active thinking and concentration. The positive correlation between these two types of waves provides insight regarding how reminiscence therapy might influence the functioning of the brains of patients with dementia [25]. Typically, α waves increase during relaxation and when the eyes are closed, but their association with β waves in this study suggests that reminiscence therapy may simultaneously promote relaxation and cognitive activity in dementia patients [26,27]. This implies that patients can actively recall memories while maintaining a relaxed state, contributing to psychological stability and stress reduction. Previous research indicates that reminiscence therapy is particularly effective in activating emotional memories, which can contribute to emotional stability and an increase in positive emotions in patients with dementia [9]. The strong correlation between α and β waves suggests that reminiscence therapy might activate both positive emotions and cognitive functions in the brains of dementia patients. This has important therapeutic implications for improving the quality of daily life of these patients. The discovery of this correlation paves the way for new methods to assess the therapeutic effects of reminiscence therapy for dementia on the basis of specific physiological markers [28]. In the future, this correlation could inform customized treatments and help maximize therapeutic effects. Further research is required, including comparative studies with other non-pharmacological interventions and studies examining the effects of reminiscence therapy across different types and stages of dementia. The findings of such studies could significantly advance the clinical application of reminiscence therapy, opening new avenues for the use of specific physiological indicators to assess therapeutic efficacy.

### 4.2. Impact of Reminiscence Therapy on Cognitive Function and Daily Activities

Reminiscence therapy’s ability to improve cognitive functions in dementia patients is suggested by the improvements seen in Hasegawa Dementia Scale scores [29,30]. In our study, although the HDS-R scores showed an improvement from a mean of 12.56 (SD 5.78) pre-intervention to a mean of 14.32 (SD 5.91) post-intervention, this change was not statistically significant (*p* = 0.078). This indicates that reminiscence therapy may be able to enhance the quality of daily life of dementia patients. However, it is important to interpret these findings cautiously, given the lack of statistical significance; the effects might not be consistent across all individuals. Although the HDS-R scores showed improvement in our patients, the lack of statistical significance necessitates cautious interpretation. The results suggest a potential benefit of reminiscence therapy in improving cognitive function but cannot be considered definitive. Therefore, there is a need for further research to confirm our findings and identify under what conditions, or in which patient subgroups, reminiscence therapy might be most effective. Additionally, comparative studies with other non-pharmacological interventions are necessary to validate the unique contributions of reminiscence therapy. These future studies could significantly enhance the clinical application of reminiscence therapy and inform evidence-based practices for dementia care.

### 4.3. Clinical Significance and Future Challenges of Reminiscence Therapy

Reminiscence therapy, as a non-pharmacological intervention, holds promise as an effective treatment option for individuals with dementia [31,32]. However, its effects can vary depending on the patient’s condition and the therapy implementation method. Future research is essential to develop detailed treatment protocols that can assess the effectiveness of different therapeutic approaches more accurately. This will clarify how the various approaches to reminiscence therapy function in the treatment of dementia. The efficacy of reminiscence therapy may vary significantly on the basis of the stage of dementia, the therapeutic environment, and the specific methods employed during sessions. Individual differences in patient responses to therapy underscore the need for personalized treatment plans that consider the unique characteristics of each patient. These protocols should be designed to standardize practices across different care settings while also allowing for adjustments on the basis of individual patient needs.

### 4.4. Strengths and Limitations

The present study has several strengths, as follows:

① Demonstration of cognitive and emotional benefits: Our findings indicate that reminiscence therapy can significantly improve the cognitive functions and emotional well-being of dementia patients. We observed measurable improvements in participants’ HDS-R scores, corroborating previous research suggesting that reminiscence therapy can enhance cognitive flexibility and emotional stability. These results affirm the therapy’s potential as a powerful intervention for dementia care.
② Advancement of non-pharmacological interventions: In the context of increasing concerns about the side effects associated with pharmacological treatments in older adults, this study reinforces the importance of non-pharmacological approaches. Reminiscence therapy, as demonstrated by our research, offers a viable alternative to drug therapies, potentially reducing the risk of adverse drug interactions and promoting a holistic approach to patient care.
③ Personalization of therapy: One of the key strengths of our study is the personalized approach taken in the administration of reminiscence therapy. By tailoring the sessions to the individual histories and preferences of each participant, we ensured high levels of engagement and relevance, which are critical for the success of psychological interventions in dementia care. This personalized approach not only supports the therapeutic process but also respects the unique life stories of each patient, thereby enhancing the therapeutic impact.

The limitations of this study are as follows:

① Variability in patient response: A significant limitation of this study is the variability in how patients responded to reminiscence therapy. The effectiveness of the therapy varied widely among participants, which may have been influenced by factors such as the stage of dementia, psychological profiles, and specific life histories of the patients. This variability poses challenges in predicting which patients will benefit most from the therapy and underscores the need for further research to identify factors predictive of success.
② Lack of standardized protocols: Our study was conducted without the use of a fully standardized protocol for reminiscence therapy, which may have affected the consistency and replicability of the findings. The absence of standardized procedures can lead to variations in therapy delivery across different settings and practitioners, potentially impacting the overall effectiveness of the intervention. Future studies should aim to develop and follow standardized protocols to enhance the reliability of the outcomes.
③ Methodological constraints: The small sample size and lack of a control group limit the generalizability of the findings. Future research should aim to include larger sample sizes and appropriate control groups to better validate the results. Additionally, comparative studies with other non-pharmacological interventions are recommended to understand the unique contributions of reminiscence therapy. Furthermore, the absence of long-term follow-up prevents us from assessing the enduring impact of the therapy, which is crucial for understanding its long-term benefits and limitations.
④ Dependence on facilitator skill: The effectiveness of reminiscence therapy relies heavily on the skill and sensitivity of the facilitators. This dependence raises concerns about the therapy’s consistency, as less experienced or inadequately trained facilitators may not achieve the same level of patient engagement or therapeutic outcomes. Ensuring that facilitators are well trained and equipped to handle the complexities of dementia care is vital for the success of the therapy.

## 5. Conclusions

This study has demonstrated that reminiscence therapy has significant potential as a non-pharmacological intervention for enhancing the cognitive functions and emotional well-being of individuals with dementia, as evidenced by improvements in HDS-R scores. These benefits underscore the therapy’s capacity to enrich patients’ lives by reconnecting them with their past experiences, thereby fostering a sense of identity and continuity. Despite its advantages, the therapy’s effectiveness varies among patients because of factors such as patient history and the stage of dementia, highlighting the need for personalized treatment approaches. Moreover, the absence of standardized protocols poses challenges in therapy delivery, affecting the consistency and replicability of the outcomes. To advance the application of reminiscence therapy in clinical settings, further research is required to develop standardized methods, understand its long-term effects, and explore its efficacy compared with other treatments. Future studies should include larger samples and control groups to enhance the robustness of the findings and support the broader implementation of reminiscence therapy in dementia care. Addressing these methodological issues will enable healthcare providers to offer more effective, personalized, and compassionate care to the growing number of individuals experiencing dementia.

## Figures and Tables

**Table 1:** Characteristics of study participants.

Variables	All		Pre- Mean (SD)	Post- Mean (SD)
	Mean (SD)	Range (Min–Max)		
EEG α	5.25 (1.41)	3.1–11.8	5.20 (1.35)	5.30 (1.48)
EEG β	2.63 (0.98)	1.3–9.7	2.61 (0.98)	2.66 (0.99)
Age	87.88 (7.66)	71–104	-	-
NPI-NH score	15.65 (15.19)	1–59	-	-
HDS-R score	12.56 (5.78)	3–29	-	-

Note: SD, standard deviation; EEG, electro encephalography; NPI-NH, Neuropsychiatric Inventory—Nursing Home Version; HDS-R, Hasegawa Dementia Scale—Revised.

**Table 2:** Factors associated with EEG β waves.

	Estimate	Std. Error	t-Value	*p*-Value
Intercept	−2.20	1.92	−1.15	0.261
EEG α waves	0.61	0.06	10.64	<0.001
Age	0.02	0.02	0.95	0.349
NPI-NH score	0.00	0.01	0.06	0.951
HDS-R score (Ref. > 20 points)	−0.31	0.32	−0.97	0.336
Number of sessions	−0.07	0.07	−1.04	0.303

Note: EEG, electro encephalography; NPI-NH, Neuropsychiatric Inventory—Nursing Home Version; HDS-R, Hasegawa Dementia Scale—Revised.

**Table 3:** Pre–post EEG α wave changes.

	Estimate	Std. Error	t-Value	*p*-Value
Intercept	2.90	2.27	1.28	0.211
Pre–post EEG changes	0.12	0.09	1.32	0.188
Number of sessions	−0.01	0.02	−0.30	0.766
Age	0.02	0.03	0.96	0.346

Note: EEG, electro encephalography.

**Table 4:** Pre–post EEG β wave changes.

	Estimate	Std. Error	t-Value	*p*-Value
Intercept	−1.35	1.33	−1.02	0.317
Pre–post EEG changes	0.08	0.08	1.00	0.320
Number of sessions	0.00	0.02	−0.07	0.945
Age	0.04	0.02	2.92	0.006

Note: EEG, electro encephalography.

## Data Availability

All data are available from the corresponding author on reasonable request.
